# Mortality Statistics

**Published:** 1875-01

**Authors:** 


***Chicago Mortality Report for November, 1874.*** Reported by Dr. BEN. C. MILLER, Sanitary Superintendent.

MORTALITY IN MONTH OF NOVEMBER.

| | | | |
|---|---|---|---|
| Abscesses | 1 | Anæmia | 1 |
| " of peritoneum | 1 | Aneurism of aorta | 1 |
| Accident, burns by kerosene | 1 | Anus imperforate | 1 |
| " crushed | 1 | Apoplexy | 2 |
| " by drowning | 1 | Asthma | 1 |
| " by fall | 3 | Bowels, intussusception | 1 |
| " thrown from buggy | 1 | Brain, abscess of | 1 |
| " by suffocation | 2 | " congestion of | 8 |
| " by railroad | 2 | " inflammation of | 4 |
| " by street cars | 2 | " irritation of | 1 |
| Abortion by self | 1 | Bronchitis | 6 |

| | |
|---|---|
| Bronchitis, capillary | 6 |
| Biliary calculi | 1 |
| Cancer | 1 |
| " of breast | 2 |
| " of bowels | 1 |
| " of liver | 2 |
| " pyloric | 1 |
| " pelvic | 1 |
| " of rectum | 1 |
| " of stomach | 2 |
| " of uterus | 4 |
| Cholera infantum | 5 |
| Consumption | 52 |
| Convulsions | 50 |
| " puerperal | 2 |
| Croup | 7 |
| " membranous | 2 |
| Cyanosis | 1 |
| Cystitis | 1 |
| Debility, general | 3 |
| Delirium tremens | 1 |
| Deficient development | 1 |
| Diabetes | 1 |
| Diarrhœa | 4 |
| " chronic | 2 |
| Diphtheria | 15 |
| Dropsy, general | 3 |
| Dysentery | 2 |
| Endo carditis | 1 |
| Enteritis | 9 |
| Entero-colitis | 2 |
| Epilepsy | 1 |
| Erysipelas | 2 |
| Exposure | 1 |
| Exhaustion | 3 |
| Fever, puerperal | 2 |
| " remittent | 1 |
| " scarlet | 11 |
| " " malignant | 1 |
| " typhoid | 25 |
| Gangrene | 1 |
| Gastritis | 2 |
| Gastro enteritis | 3 |
| Hemorrhage funis | 1 |
| Hemorrhagica purpura | 1 |
| Heart, aneurism of | 1 |
| " disease of | 4 |
| " enlargement of | 2 |
| " organic disease of | 1 |
| Heart, rheumatism of | 1 |
| " valvular disease of | 6 |
| Hemiplegia | 1 |
| Hepatitis | 1 |
| Hernia | 1 |
| " strangulated | 1 |
| Hydrothorax | 1 |
| Hydrocephalus | 6 |
| Inanition | 5 |
| Intemperance | 2 |
| Jaundice | 1 |
| Kidneys, disease of | 1 |
| " and bladder, disease of | 1 |
| " Bright's disease of | 3 |
| Laryngitis | 1 |
| Liver, congestion of | 1 |
| " induration of | 1 |
| " stricture of | 1 |
| Lungs, congestion of | 9 |
| " apoplexy of | 2 |
| " hemorrhage of | 1 |
| " gangrene of | 1 |
| " œdema of | 1 |
| " abscess of | 2 |
| Mania, puerperal | 1 |
| Measles | 1 |
| Meninges, inflammation of | 1 |
| Meningitis | 6 |
| " cerebro-spinal | 8 |
| Metritis | 1 |
| Old age | 15 |
| Paralysis | 2 |
| Peritonitis | 3 |
| Pneumonia | 24 |
| " typhoid | 1 |
| Rupture of aorta | 1 |
| Scrofula | 2 |
| Suicide, by laudanum | 1 |
| " poison | 2 |
| Syphilis | 1 |
| Tabes mesenterica | 15 |
| Teething | 1 |
| Tumor | 1 |
| Uterus, hemorrhage of | 1 |
| Vitality, deficient | 1 |
| Whooping cough | 4 |
| Total | 436 |

Premature births, 7; Still births, 61. Total 68

COMPARISON.

| | | |
|---|---|---|
| Deaths in month of November, 1874 | | 436 |
| " " October, 1874 | | 570 |
| Decrease | 134 | |
| Deaths in month of November, 1873 | | 549 |
| Decrease | 113 | |

AGES.

| | | | |
|---|---|---|---|
| Under one year | 119 | Thirty years to forty | 45 |
| One year to two | 36 | Forty " " fifty | 31 |
| Two years to three | 26 | Fifty " " sixty | 21 |
| Three " " four | 8 | Sixty " " seventy | 25 |
| Four " " five | 2 | Seventy " " eighty | 21 |
| Five " " ten | 23 | Eighty " " ninety | 5 |
| Ten " " twenty | 23 | Ninety " " one hundred | 3 |
| Twenty " " thirty | 48 | Total | 436 |
| Males | 233 | Colored | 7 |
| Females | 203 | White | 429 |
| Total | 436 | Total | 436 |

Married, 164; Single, 272. Total 436

NATIVITIES.

| | | | |
|---|---|---|---|
| Belgium | 2 | Holland | 3 |
| Bohemia | 5 | Ireland | 43 |
| Canada | 6 | Norway | 5 |
| Native—Chicago | 46 | Nova Scotia | 2 |
| Foreign, " | 137 | Poland | 1 |
| United States, other parts | 87 | Scotland | 2 |
| Denmark | 2 | Sweden | 7 |
| England | 15 | Switzerland | 1 |
| France | 1 | Wales | 2 |
| Germany | 64 | Unknown | 5 |
| | | Total | 436 |

Deaths daily, 14½. Mean temperature, 40.4°. Rain fall, 2.72 inches.

MORTALITY BY WARDS.

| Wards. | No. Deaths. | Pop. in 1874. | Percentage. |
|---|---|---|---|
| 1 | 5 | 5,725 | one death in 1,145 |
| 2 | 1 | 4,830 | " " 4,830 |
| 3 | 15 | 14,861 | " " 991 |
| 4 | 14 | 15,361 | " " 1,097 |
| 5 | 18 | 20,078 | " " 1,115 |
| 6 | 47 | 35,916 | " " 764 |
| 7 | 35 | 31,722 | " " 906 |
| 8 | 28 | 29,143 | " " 1,041 |
| 9 | 34 | 31,654 | " " 931 |
| 10 | 11 | 17,385 | " " 1,580 |
| 11 | 9 | 14,022 | " " 1,558 |
| 12 | 11 | 16,792 | " " 1,527 |
| 13 | 14 | 17,892 | " " 1,277 |
| 14 | 12 | 16,720 | " " 1,338 |
| 15 | 59 | 45,545 | " " 772 |
| 16 | 23 | 21,922 | " " 953 |
| 17 | 17 | 20,777 | " " 1,234 |
| 18 | 21 | 21,392 | " " 1,019 |
| 19 | 4 | 4,677 | " " 1,169 |
| 20 | 7 | 8,995 | " " 1,285 |
| | 385 | Ratio of deaths to population in 1874, one death in 907. | |

| | | | |
|---|---|---|---|
| No. deaths in Wards | 385 | Old Ladies' Hospital | 1 |
| Accidents | 13 | St. Joseph's Hospital | 1 |
| Alexian Bros. Hospital | 1 | St. Luke's Hospital | 2 |
| County Hospital | 9 | Suicides | 3 |
| Foundlings' Home | 15 | Woman's Hospital | 1 |
| Hospital for Women and Child'n. | 2 | | |
| Mercy Hospital | 3 | Total | 436 |

